# Supplementary material for: Jamming with magnetic composites
Source: Nat Commun. 2025 Sep 30;16:8711. doi: 10.1038/s41467-025-63729-z (PMC12484853; doi:10.1038/s41467-025-63729-z)
Supplement: Supplementary file 1 — Supplementary Information [file 41467_2025_63729_MOESM1_ESM.pdf]

Jamming with Magnetic Composites
Supplementary Information

Buse Aktaş, Minsoo Kim, Marc Bäckert, Gianluca Sicilia, Gian-Luca Franchini, Florian Heemeyer, Simone Gervasoni, Xiang-Zhong Chen, Salvador Pané, Bradley J. Nelson

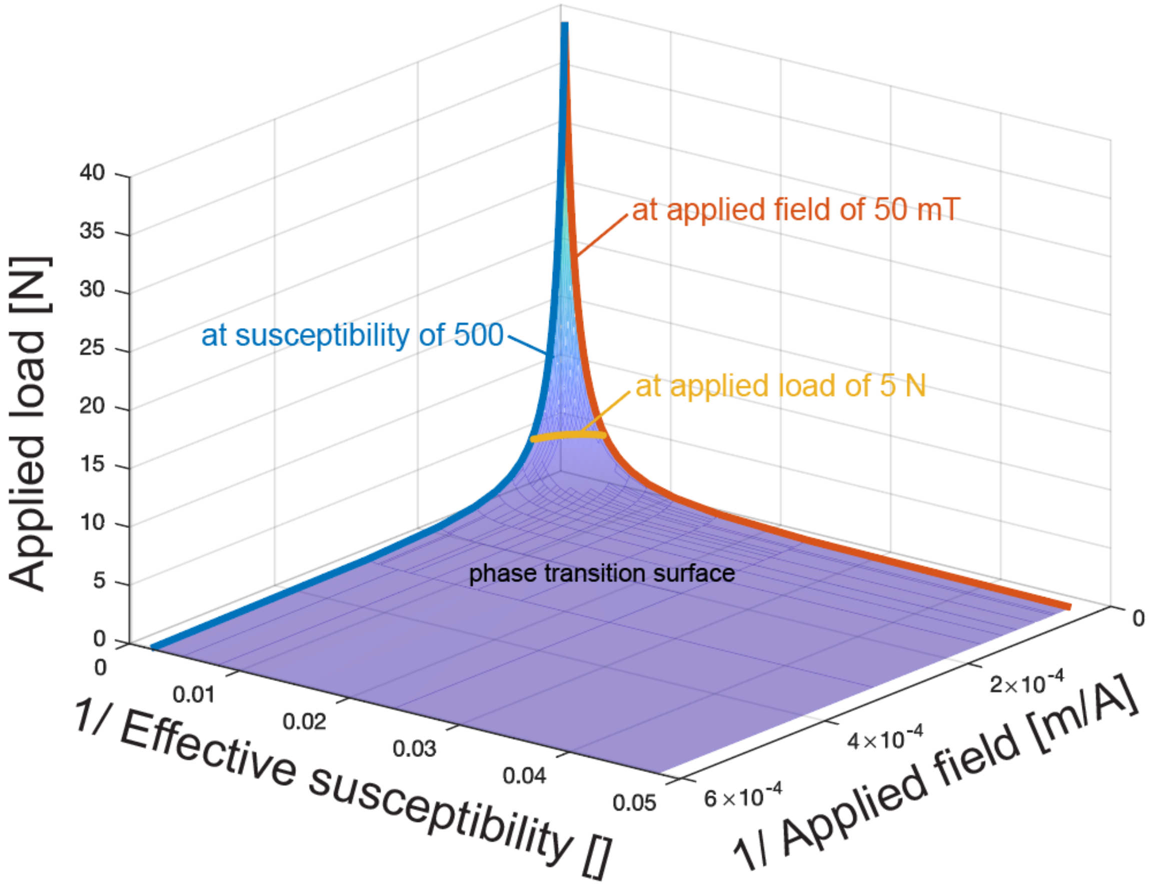

Supplementary Figure 1 | Magnetic jamming phase diagram: It was shown that the jamming transition load is governed by the localized breakaway, slip, or separation forces which occur between two adjacent magnetic composite subunits in pivoting, sliding, or separating modes, respectively. This load is calculated as product of a constant and the attraction force between two subunits. The constant depends on geometric entities for breakaway, is equal to the friction coefficient for slip (assuming a simple Coulomb model of friction) and is a value of 1 for separation. A simplified model of the attraction force, assuming each subunit is a dipole with effective susceptibility of χ_{eff} and volume of V, can be utilized, where an applied magnetic field of H and a dipole-dipole distance of d is also taken into account: $\mathrm{F}_{attraction} = \frac{\mu_0 H^2 (V\chi_{eff})^2}{4\pi d^4}$. The resulting phase diagram is quantitatively shown above. In this phase diagram, the magnetic volume was kept constant at V = 1 mm^3, the distance between the two dipoles was taken to be d = 1 mm, and the attraction force was not multiplied by a constant.

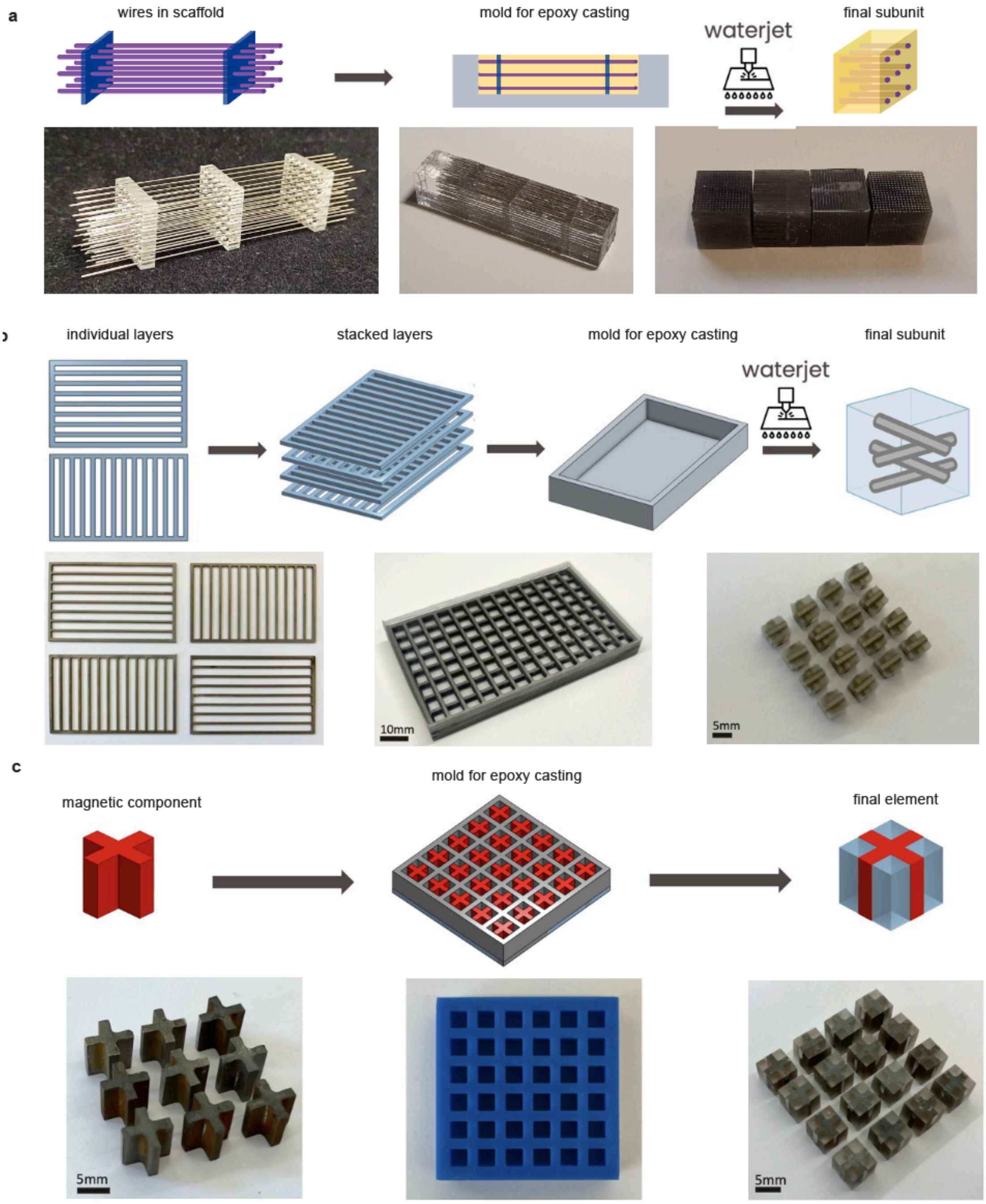

Supplementary Figure 2 | Fabrication of composite subunits for magnetic jamming: a. The 1-D jamming structures were fabricated using laser-cut acrylic scaffolds to ensure programmable pillar spacing. The wires were placed in the scaffold and then in a silicone mold for epoxy casting. Then, the entire composite block was cut into smaller subunits using a waterjet. **b.** The 2-D jamming structures were fabricated using a layer-by-layer fabrication method. Each layer was patterned with a waterjet, and then stacked into a silicone mold for epoxy casting. The resulting block was also waterjet into individual subunits. **c.** The 3-D jamming structures were fabricated as individual subunits. The magnetic part of each subunit was prepared using a water jet, they were then placed into individual molds. The resulting casts are the final subunits.

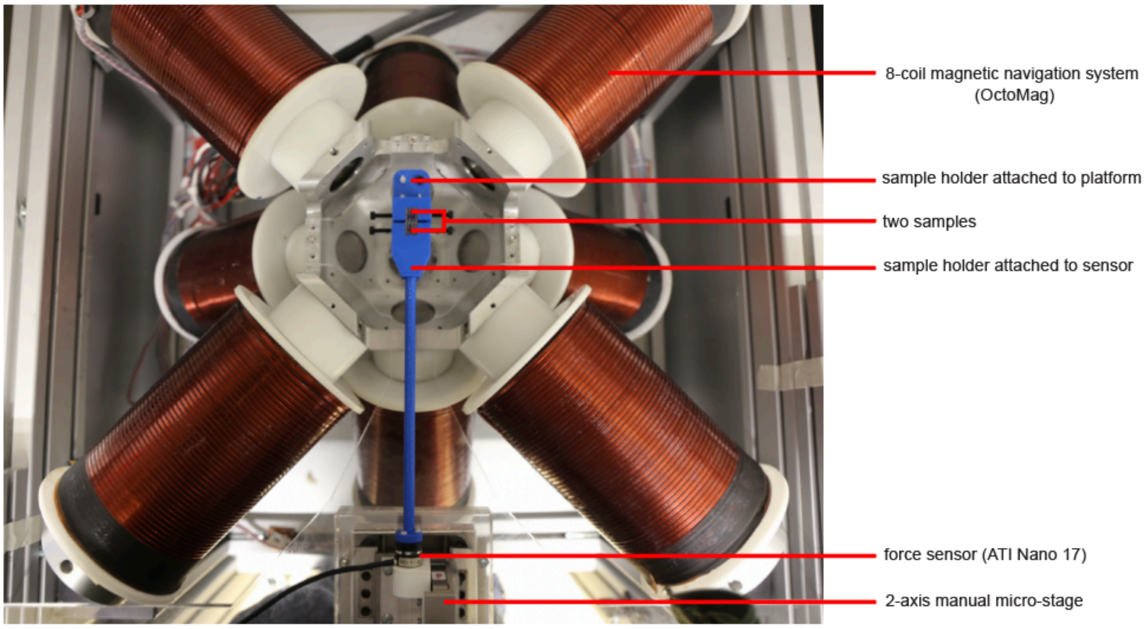

Supplementary Figure 3 | Experimental setup to measure inter-subunit attractive forces: A force-measurement setup within the magnetic workspace of the OctoMag was used to determine forces. While one sample was fixed to the main platform, the other was attached to a high precision sensor (ATI Nano 17) and two linear stages, which enabled manual alignment.

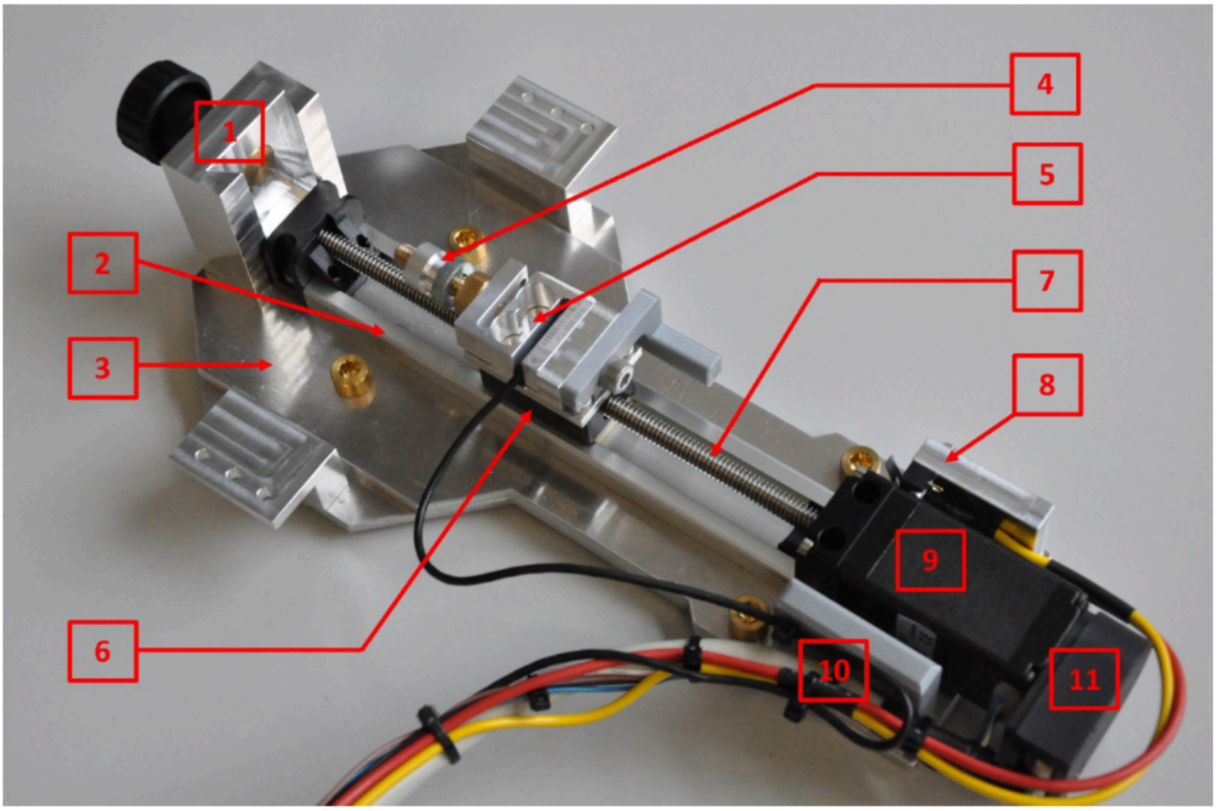

Supplementary Figure 4 | Mechanical characterization device to extract force-displacement curves for magnetic jamming structures: A custom device was fabricated such that displacement could be controlled and force could be measured in any type of sample without being influenced by the surrounding magnetic fields. (1) A dovetail fixture for sample holders allows multiple types of tests to be conducted (shear, bending, etc.). (2) The main body of the linear motion system is aluminum. (3) An aluminum plate holds all elements. (4) Threaded holding screw with locking nut allows for mounting of special tools for diverse experiments. (5) A force sensor is mounted on (6) the carriage, which is moved along the aluminum fixture by the lead screw. The actuation system consists of (7) an aluminum lead screw driven by a stepper motor, (8) a micro switch assisting with homing, and (9) a stepper motor. (10) A cable support hub minimizes damage to the pins and plugs that connect the wires to the device. (11) A relative encoder assists in accurate movement control of the motor by sending feedback to driver for closed-loop control.

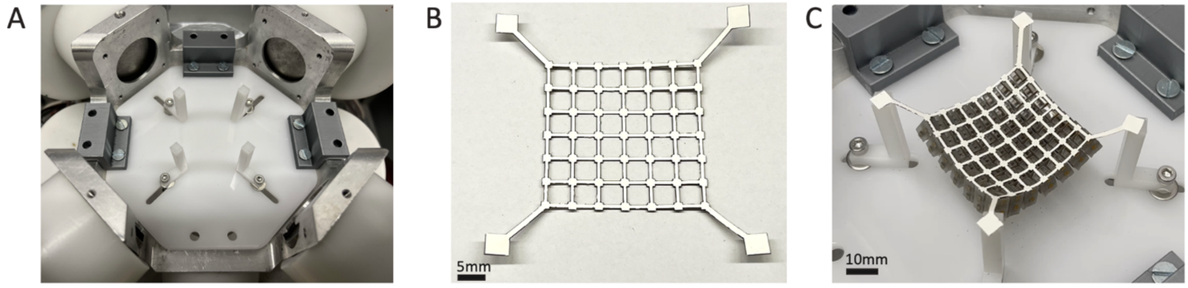

Supplementary Figure 5 | Fabrication process and experimental setup for 2-D stiffness tunable structures: A. An experimental setup was fabricated to suspend the two-dimensional structure from its four corners, such that non-zero Gaussian curvatures could be observed in the beam enabling independent bending along two directions. **B.** The strain limiting layer was fabricated as a grid-like pattern to enable multi-axial deformation of each subunit in relation to its neighboring units. It was fabricated from a tape sheet (B4775-50, Avery Zweckform) using a laser cutter. **C.** The structure is shown suspended from the experimental setup situated within the magnetic navigation system utilized.

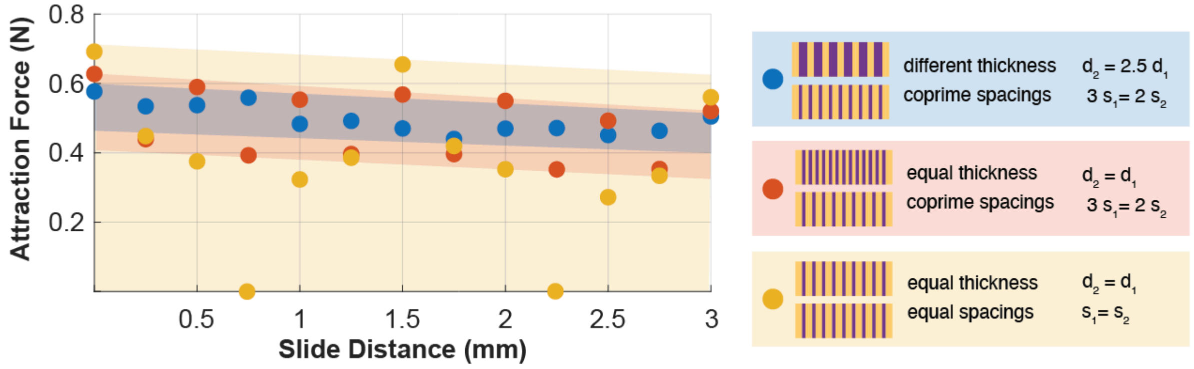

Supplementary Figure 6 | Composite design influence on force profile during the sliding deformation mode. Simulations were conducted to demonstrate a methodical and effective strategy to design for the oscillatory behavior during the sliding mode, and these were compared with the standard homogeneous composite design (in yellow). The magnetic material was redistributed inside the composite, without changing the overall magnetic volume, and the attraction between two neighboring subunits was extracted from simulation results. More specifically: a coprime relative distribution between neighboring subunits, helps avoid points where the two sides have fully matching or fully out of phase patterns, and ensures consistent overlap throughout deformation regime (in red). The oscillation can be further reduced by keeping the spacings coprime, while having different pillar thicknesses in neighboring subunits (in blue).
