# Supplementary material for: Jamming with magnetic composites
Source: Nat Commun. 2025 Sep 30;16:8711. doi: 10.1038/s41467-025-63729-z (PMC12484853; doi:10.1038/s41467-025-63729-z)
Supplement: Supplementary file 2 — Description of Additional Supplementary Files [file 41467_2025_63729_MOESM2_ESM.pdf]

Description of Additional Supplementary Files

Supplementary Movie 1
Funnel experiments with linear, planar, and volumetric magnetic jamming structures demonstrate how the structures can withstand loads and retain their shape when a magnetic field is applied, but immediately collapse when the magnetic field is removed.

Supplementary Movie 2
Lifting experiments demonstrate that individual subunits of a magnetic jamming structure remain independent when there is no external magnetic field but become cohesive with their neighboring subunits when a magnetic field is applied.

Supplementary Movie 3
Representative mechanical characterization tests demonstrating the force-deformation behavior induced by the sliding or pivoting modes between two magnetic jamming subunits.

Supplementary Movie 4
Representative mechanical characterization tests of magnetic jamming-based beams in different boundary conditions, first in a simply supported three-point bending configuration and then in a double clamped configuration.

Supplementary Movie 5
The multi-degree-of-freedom assembly and jamming of a two-dimensional magnetic jamming structure is demonstrated by showing that it can be jammed in either axis or simultaneously in both axes.

Supplementary Movie 6
Lifting experiments demonstrate that jamming can be independently controlled in all three-degrees-of-freedom by simply changing the direction of the applied magnetic fields.

Supplementary Movie 7
Multi-dimensional stiffness control is demonstrated in a magnetic jamming-based sheet, which can be stiffened in either axis or simultaneously in both axes.

Supplementary Movie 8
A magnetic composite-based gripper demonstrates a tunable gripping force, and independent control of the motion and orientation.

Supplementary Movie 9
A dilation beam which can either conform to a soft flexible environment, or can stiffen and apply a force to this environment is demonstrated.

Supplementary Movie 10
Distributed jamming demonstrates that the subunits need not be one single structure, but can be distributed into smaller sub-structures in a complex environment, and can be controlled simultaneously.
